# A Food Matrix Triggers a Similar Allergic Immune Response in BALB/c Mice Sensitized with Native, Denatured, and Digested Ovalbumin

**DOI:** 10.3390/life13081733

**Published:** 2023-08-12

**Authors:** Jesús Gilberto Arámburo-Gálvez, Raúl Tinoco-Narez-Gil, Aldo Alejandro Arvizu-Flores, Oscar Gerardo Figueroa-Salcido, José Antonio Mora-Melgem, Alma Rosa Islas-Rubio, Lilian Karem Flores-Mendoza, Veronica Lopez-Teros, Humberto Astiazaran-Garcia, Feliznando Isidro Cárdenas-Torres, Noé Ontiveros

**Affiliations:** 1 Graduate Program in Health Sciences, Faculty of Biological and Health Sciences, Universidad de Sonora, Hermosillo 83000, Sonora, Mexico; gilberto.aramburo@uas.edu.mx (J.G.A.-G.); aldo.arvizu@unison.mx (A.A.A.-F.); veronica.lopez@unison.mx (V.L.-T.); hastiazaran@ciad.mx (H.A.-G.); 2 Nutrition Sciences Graduate Program, Faculty of Nutrition Sciences, Autonomous University of Sinaloa, Culiacan 80019, Sinaloa, Mexico; raul.narez.uacng@uas.edu.mx (R.T.-N.-G.); oscar.figueroa@uas.edu.mx (O.G.F.-S.); josemora.uacng@uas.edu.mx (J.A.M.-M.); 3 Integral Postgraduate Program in Biotechnology, Faculty of Chemical and Biological Sciences, Autonomous University of Sinaloa, Ciudad Universitaria, Culiacan 80010, Sinaloa, Mexico; 4 Department of Plant-Origin Food Technology, Research Center for Food and Development, CIAD, A.C. Carretera Gustavo Enrique Astiazarán Rosas, No. 46 Col. La Victoria, Hermosillo 83304, Sonora, Mexico; aislas@ciad.mx; 5 Clinical and Research Laboratory (LACIUS, C.N.), Department of Chemical, Biological, and Agricultural Sciences (DC-QB), Faculty of Biological and Health Sciences, University of Sonora, Navojoa 85880, Sonora, Mexico; lilian.flores@unison.mx; 6 Department of Nutrition, Research Center for Food and Development, CIAD, A.C. Carretera Gustavo Enrique Astiazarán Rosas, No. 46 Col. La Victoria, Hermosillo 83304, Sonora, Mexico

**Keywords:** food allergy, food matrix, allergic reaction, ovalbumin

## Abstract

The search for an animal model to evaluate the allergenic potential of processed food products is still ongoing. Both the sensitization to ovalbumin (OVA) in different structural states and the allergic response triggered after intragastric or food challenges were assessed. BALB/c mice were sensitized intraperitoneally to OVA (50 µg) in different structural states (native OVA, N-OVA; denatured OVA, D-OVA; formaldehyde- and lysine-treated OVA, FK-OVA; denatured OVA-FK, OVA-DFK; peptides from pepsin digestion, Pep-OVA). Anti-OVA-specific IgE responses were evaluated using ELISA. Anaphylactic signs and mMCP-1 serum levels were evaluated after intragastric (2.0 mg/OVA) and food (0.41 mg/OVA) challenges. IgE reactivities to N-OVA and D-OVA were similar among groups (*p* > 0.05). After the challenges, all OVA-sensitized mice developed mild to severe anaphylactic signs (*p* < 0.05 vs. control). Mice sensitized to N-OVA and D-OVA had the highest mMCP-1 serum levels after challenges (*p* < 0.05 vs. control). Allergic responses were similar despite the different OVA doses used for the challenges. The N-OVA-sensitized murine model of egg allergy proposed in the present study holds the potential for evaluating the impact of food matrix composition and processing on the threshold of egg-allergic responses.

## 1. Introduction

Allergenic components are part of many processed foods and can interact with other macronutrients during food processing [1]. These molecular interactions can diminish or abolish the potential of food allergens to trigger allergic responses [2,3] and even affect the performance of conventional methods for the detection and quantification of allergens [1]. An animal model of food allergy can be a key tool for evaluating with a high degree of certainty not only the safety of processed food products for allergic people, but also the safety of transgenic foods for consumers [4]. Notably, most food allergy rodent models imply sensitization and allergic elicitation with the allergen of interest in its native state [4,5], leaving aside the sensitizing and allergenic potential of allergens in different structural states. The interactions of allergens with the food matrix components and/or the structural modifications induced during gastrointestinal digestion have the potential to trigger an allergic response to food allergens [4]. Here, we present a novel approach to better understanding how food allergy animal models become sensitized or trigger an allergic immune response after the administration of protein allergens in different structural states, laying the groundwork to develop rodent models with the capability to evaluate the allergenic potential of processed foods. For instance, it remains unknown whether the intraperitoneal (IP) sensitization of mice with proteins in different structural states can trigger a differential allergic immune response after an oral challenge with the food matrix containing the allergen of interest. Therefore, in the present study, we assessed both the capability to IP sensitize BALB/c mice with ovalbumin (OVA) in various structural states and the potential to trigger in the sensitized mice an allergic response after a food challenge with a matrix containing OVA.

## 2. Materials and Methods

### 2.1. Materials

Chicken egg white ovalbumin (Sigma, Saint Louis, MO, USA, CAS: 9006-59-1), phosphate-buffered saline (PBS) (8.0 g NaCl, 1,16 g Na_2_HPO_4_, 0.2 g KH_2_PO_4_, 0.2 g KCl; 1 L, pH 7.4), phosphate buffer (PB) (0.1 M NaH_2_PO_4_ and 0.1 M Na_2_HPO_4_, pH 7.4), simulated gastric fluid (SGF) (HCL 0.0084 N, NaCl 35 mM, pH 1.3), Imject^®^ Alum (Thermo Scientific, Rockford, IL, USA, product code 77161), formaldehyde solution (Sigma-Aldrich, Saint Louis, MO, USA, CAS-No: 50-00-0), lysine (Sigma-Aldrich, Saint Louis, MO, USA, CAS: 56-87-1), pepsin (Sigma-Aldrich, CAS: 9001-75-6), Tween 20 (Faga Lab^®^, Mocorito, Sinaloa, Mexico, product code 2377), egg (Ovalbumin) ELISA Kit (Morinaga, Tsurumi-ku, Yokohama-shi, Japan, Cat.# M2101), mMCP1 ELISA kit (BioLegend, San Diego, CA, USA, Cat. 432702), PierceTM BCA protein assay kit (Thermo Scientific, #23225), laboratory rodent diet (LabDiet 5001, St. Louis, MO, USA), polypropylene feeding tubes (Instech Laboratories, Inc., Cat. 20 GA × 38 mm), biotinylated anti-mouse IgEa antibody (BioLegend, San Diego, CA, USA, Cat. 408804), 96-wells plates (Costar Assay plate, EE. UU), streptavidin-horseradish peroxidase (BioLegend. Cat. 405210), and tetramethyl benzidine TMB (Thermo Scientific, Cat. 34028) were used in the present study.

### 2.2. Animals

A total of 36 pathogen-free female BALB/c mice, aged 5 to 6 weeks, was used in this study (acquired from BIOINVERT, Estado de México, México). Mice were housed in an animal facility under controlled pathogen-free conditions, with a relative humidity of 40–60%, a temperature of 23 ± 2 °C, and 12-h light and dark cycles. Throughout the experiments, mice were fed an egg- and ovalbumin-free diet (LAbDiet 5001), with water and food provided ad libitum. The ethics review board of the Autonomous University of Sinaloa approved the study protocol. Ethical approval number: CE-UACNyG-2014-JUL-001.

### 2.3. Preparation of Food Matrix Model

Baked cookies were utilized as a food matrix model. The cookies were prepared according to the AACC Method 10–50D with minor modifications [6]. Briefly, wheat flour containing 1000 ppm of OVA was used to simulate potential interactions between the food matrix ingredients and the allergen during the cookie preparation. The cookie formulation was as follows: wheat (*Triticum aestivum*) flour with OVA at 1000 ppm (220.1 g), butter (65 g), sugar (130 g), salt (2.1 g), sodium bicarbonate (2.5 g), water (20.9 mL), and dextrose (33 g of dextrose solution at 5.93 g/mL). The concentration of OVA in the baked cookies was determined using the commercial egg (Ovalbumin) ELISA Kit II (Morinaga Institute of Biological Science, Yokohama, Japan), following the manufacturer’s instructions.

### 2.4. Antigen Preparation for Sensitization Protocols

Different forms of OVA were prepared according to Koch et al. [7] and Holm et al. [8], with slight modifications. Native OVA (N-OVA) was dissolved in PBS (1 mg/mL) and filter-sterilized (0.22 μm) before use. Denatured OVA (D-OVA) was prepared by dissolving OVA in PB (1 mg/mL) and autoclaving at 110 °C for 1 h. This solution was centrifugated at 10,000× *g* for 30 min, and the supernatant was dialyzed against PBS in a 500 Da membrane. The dialyzed solution was stored at −80 °C and filter-sterilized (0.22 μm) before use.

A negative charge was conferred on OVA by treating it with formaldehyde and lysine (FK-OVA). This treatment prevents OVA aggregations and precipitations that may occur during heat treatment [8]. FK-OVA is expected to retain its immunogenic and antigenic properties, which are crucial characteristics for stimulating antibody production and proper antigen recognition [8]. OVA was dissolved in PB (1 mg/mL), and 35% formaldehyde was added to achieve a concentration of 0.025 M. Lysine was added to achieve a final concentration of 0.025 M. This solution was incubated for 2 weeks at 35 °C with constant and gentle agitation. Subsequently, the solution was centrifugated and dialyzed as described above. A fraction of FK-OVA was autoclaved, centrifuged, and dialyzed as mentioned above (Denatured FK-OVA (DFK-OVA)).

An OVA peptide solution (Pep-OVA) was obtained according to Thomas et al., with some modifications [9]. Briefly, a solution of OVA (N-OVA, 5.0 mg/mL) was mixed with simulated gastric fluid (ratio 1:20, OVA solution: SGF) containing 3 U of pepsin activity/μg of OVA. The solution was then gently shaken for 1 h in a water bath at 37 °C. A solution of 200 mM NaHCO_3_, pH 11.0 (1:3 ratio V/V) was used to stop the reaction. Finally, the peptides with a molecular weight less than 5 kDa were separated through a 5 kDa centrifugal filter, according to the manufacturer’s instructions (Amicon Ultra 15 mL Centrifugal Filters). Protein concentrations were determined using the BCA assay kit following the manufacturer’s instructions (Thermo Scientific, #23225).

### 2.5. Sensitization of Mice

**Figure 1:**
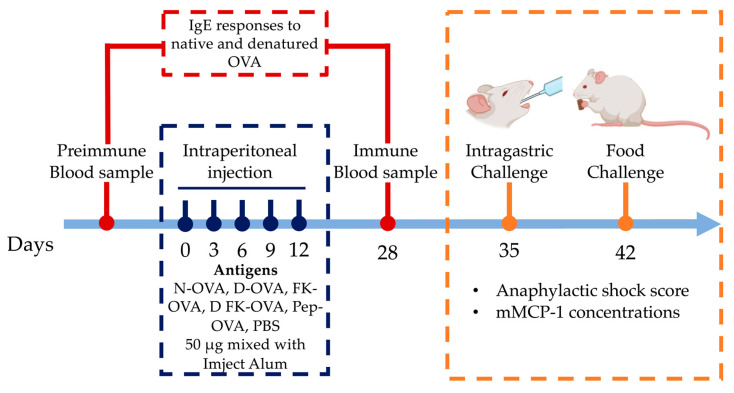
Sensitization protocol and intragastric/oral food challenge. Mice were IP sensitized to different forms of OVA or PBS using Imject Alum as an adjuvant. On days 35 and 42, the anaphylactic shock score and mMCP-1 levels were determined after intragastric (N-OVA) or oral food challenges (Cookie), respectively. Acronyms used: N-OVA: native ovalbumin; D-OVA: denatured ovalbumin; FK-OVA: formaldehyde and lysine-treated ovalbumin; DFK-OVA: formaldehyde and lysine-treated denatured ovalbumin; Pep-OVA; ovalbumin peptides; PBS: phosphate-buffered saline; mMCP-1: mouse mast cell protease-1.

The IP sensitization route induces more robust IgE responses against OVA than the oral/intragastric one [10]. Thus, a 28-day IP sensitization protocol with adjuvant was used (Imject Alum, Thermo Scientific, aluminum hydroxide 40 mg/mL; magnesium hydroxide 40 mg/mL) [11] (Figure 1). Mice were randomized into six experimental groups (n = 6). The treatments were as follows: (1) N-OVA, (2) D-OVA, (3) FK-OVA, (4) D FK-OVA, (5) Pep-OVA, and (6) Control (PBS). IP administrations (50 μg of antigen in 100 μL of PBS mixed with 100 μL Imject Alum) were carried out on days 0, 3, 6, 9, and 12. The control group received PBS only. Blood samples were collected 2 days before (preimmune sample) and 28 days after (immune sample) the first IP injection (day 0). Blood samples were centrifuged at 6000 rpm at room temperature for 15 min, and serum samples were collected and stored at −80 °C until their use for IgE antibody analysis.

### 2.6. Serum IgE Titers Evaluation

The serum IgE reactivity to N-OVA and D-OVA was evaluated using ELISA [10,11,12]. 96-well plates were coated with either 20 µg of N-OVA or D-OVA in 100 µL of coating buffer (50 mM NaHCO_3_, pH 9.6) and incubated for 16 h at 4 °C. The wells were washed three times with 200 μL of washing solution (PBS/0.05% Tween-20, pH 7.4) and blocked for 2 h with 200 μL of diluent solution (10% fetal bovine serum in PBS). After washing, 100 µL of serum samples (diluted 1:10 with diluent solution) was added and incubated for 16 h. The plates were then washed, and 100 µL of biotinylated anti-mouse IgEa antibody (diluted 1:250 in diluent solution; 2 μg/mL) was added and incubated at room temperature for 1 h. After the incubation and washing, 100 µL of streptavidin-horseradish peroxidase (diluted 1:250 in the diluent solution) was added and incubated at room temperature for 30 min. Plates were washed six times, and the TMB substrate was added (100 µL per well). The reaction was stopped by adding 50 µL of 2N H_2_SO_4_ after 30 min incubation. The optical density was measured at 450 nm. and the results were presented as absorbance fold-change, as previously described [10].

### 2.7. Intragastric and Oral Food Challenge

One week after the immune blood samples were collected (day 35), mice were challenged with 2.0 mg of N-OVA in 250 μL of PBS (intragastric challenge) (Figure 1). The challenge was carried out after 12 h of fasting using polypropylene feeding tubes (FTP-20-38, 20 GA × 38 mm) [12]. Mice were immediately placed in individual cages after the challenge, and blinded observers (n = 6) recorded for up to 30 min the severity of allergic signs using a validated anaphylactic score [13]. The score was as follows (0 to 5): 0: no symptoms; 1: scratching nose and mouth; 2: swelling around the eyes and mouth, piloerection, reduced activity, increased breathing rate; 3: shortness of breath, cyanosis around mouth and tail, increased breathing rate; 4: unresponsive to stimulation, shivering, and muscle contractions; 5: death by shock. Mice were bled 30 min after the challenges, and serum samples were obtained and stored at −80 °C until mouse mast cell protease-1 (mMCP-1) analysis was carried out.

On day 42, food challenges were carried out (Figure 1). After 12 h of fasting, mice were placed in individual cages, and 1.0 g of a cookie prepared with flour containing OVA at 1000 ppm was provided (0.410 mg of OVA per cookie portion as estimated by the egg (Ovalbumin) ELISA kit). The cookies were consumed freely for 60 min, and then blood samples were collected. The cookie leftovers were weighted to estimate consumption. The severity of allergic signs was evaluated as described above. The mMCP-1 concentrations in serum from intragastric- and oral-challenged mice were determined using a commercial ELISA kit according to the manufacturer’s instructions.

### 2.8. Statistical Analysis

Data are presented as the median and interquartile range or mean and standard deviation, depending on the data distribution (evaluated by the Shapiro–Wilk test). The Grubbs’ test was used to identify outliers. Differences between groups were analyzed using the Kruskal–Wallis test, followed by a Dunn’s test. Differences between paired values were determined using the Wilcoxon test. A *p* value < 0.05 was considered statistically significant. All analyses were performed using GraphPad Prism Version 9.0 (GraphPad Software, San Diego, CA, USA).

## 3. Results

### 3.1. IgE Responses against Native and Denatured Ovalbumin

**Figure 2:**
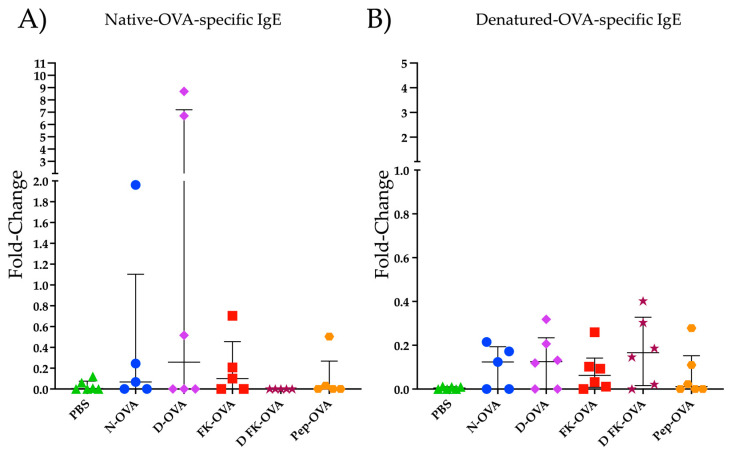
IgE reactivity to N-OVA and D-OVA. (**A**) IgE reactivity to N-OVA was expressed as fold-change. (**B**) IgE reactivity to D-OVA was expressed as fold-change. The data are presented as the median and interquartile range. Acronyms used: PBS: phosphate-buffered saline; N-OVA: native ovalbumin; D-OVA: denatured ovalbumin; FK-OVA: formaldehyde and lysine-treated ovalbumin; DFK-OVA: formaldehyde and lysine-treated denatured ovalbumin; Pep-OVA; ovalbumin peptides. The interquartile range is represented by the lines. Each experimental group is denoted by a distinct shape: PBS by a triangle, N-OVA by a circle, D-OVA by a diamond, FK-OVA by a square, DFK-OVA by a star, and pep-OVA by a hexagon.

Figure 2 shows the IgE reactivity to N-OVA and D-OVA. In the N-OVA, D-OVA, and FK-OVA sensitized groups, three to four out of six mice had IgE-positive responses against N-OVA (Figure 2A). In the DFK-OVA and Pep-OVA groups, only one and two out of six animals had an IgE positive response against N-OVA, respectively (Figure 2A). Statistical differences were not found among these experimental groups (*p* > 0.05). Regarding D-OVA, IgE reactivities against this antigen were more consistent but less robust than the ones detected against N-OVA (Figure 2A,B). No statistical differences were observed among the experimental groups in the D-OVA-specific evaluations (*p* > 0.05) (Figure 2B).

### 3.2. Intragastric and Oral Food Challenge Evaluations

**Figure 3:**
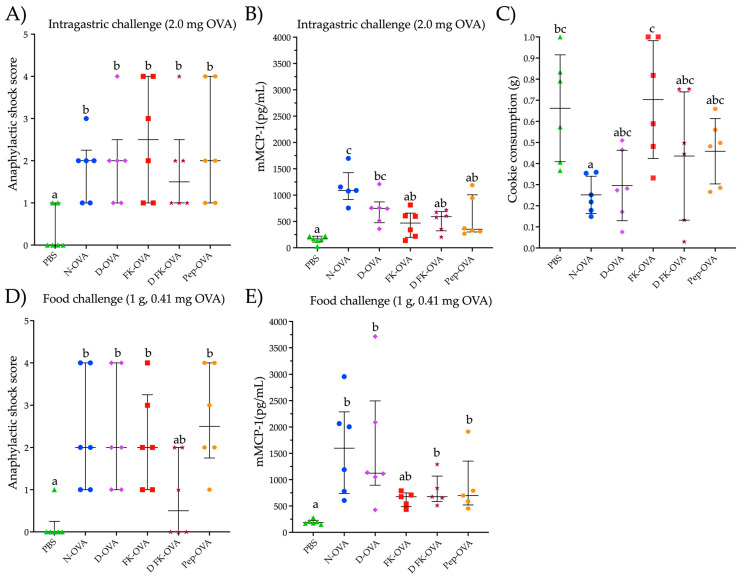
Parameters evaluated after intragastric or oral food challenges. (**A**) Anaphylactic shock score after an intragastric challenge with 2.0 mg of N-OVA. (**B**) mMCP-1 serum levels after 30 min of an intragastric challenge with 2.0 mg of OVA. (**C**) Consumption of the cookie after 60 min of exposure to it. (**D**) Anaphylactic shock score after a food challenge with a cookie containing 0.41 mg of OVA. (**E**) mMCP-1 serum levels after 60 min of a food challenge with a cookie containing 0.410 mg of OVA. Data are presented as median and interquartile range in the (**A**,**B**,**D**,**E**) sections, and as median and standard deviation in the (**C**) section. Different letters indicate statistical differences (*p* < 0.05). Acronyms used: OVA: ovalbumin; PBS: phosphate-buffered saline; N-OVA: native ovalbumin; D-OVA: denatured ovalbumin; FK-OVA: formaldehyde and lysine-treated ovalbumin; DFK-OVA: formaldehyde and lysine-treated denatured ovalbumin; Pep-OVA; ovalbumin peptides; mMCP-1: mouse mast cell protease-1. Each experimental group is denoted by a distinct shape: PBS by a triangle, N-OVA by a circle, D-OVA by a diamond, FK-OVA by a square, DFK-OVA by a star, and pep-OVA by a hexagon.

To assess the potential for triggering an allergic response to OVA, mice that were sensitized with different forms of OVA underwent an intragastric challenge with a N-OVA solution (2.0 mg OVA in 250 μL) and a food challenge with a cookie containing OVA. After the intragastric challenge, all mice sensitized to any form of OVA developed mild to severe anaphylactic shock signs (score range 1–4) (Figure 3A). The anaphylactic responses were higher in all the OVA-sensitized groups compared to the control group (PBS group) (*p* < 0.05) (Figure 3A). No statistical differences were observed in the anaphylactic score among the OVA-sensitized groups. Notably, all the mice showed detectable mMCP-1 serum levels (Figure 3B). In particular, the mice sensitized with N-OVA or D-OVA exhibited the highest mMCP-1 concentrations (*p* < 0.05, compared to PBS).

Food-challenged mice consumed between 0.25 g and 0.70 g of a cookie containing 410 ppm of OVA (ingestion of 0.10 mg to 0.27 mg of OVA) (Figure 3C). The FK-OVA-sensitized group consumed the largest cookie portion (0.70 ± 0.28 g (0.30 ± 0.11 mg of OVA)) followed by the PBS-sensitized group (0.66 ± 0.25 g (0.27 ± 0.10 mg of OVA)). The N-OVA- and D-OVA-sensitized groups registered the lowest cookie consumption (between 0.25 ± 0.08 g and 0.29 ± 0.16 g (0.10 ± 0.03 and 0.12 ± 0.06 mg of OVA, respectively)). All mice sensitized to N-OVA, D-OVA, FK-OVA, and Pep-OVA developed mild to severe anaphylactic signs after cookie consumption (scores ranged from 1 to 4) compared to the PBS control group (*p* < 0.05) (Figure 3D). With regard to the D-FK-OVA group, only two out of six mice developed mild signs of anaphylactic shock after the food challenge (Figure 3D). In line with the intragastric challenge, all OVA-sensitized groups presented detectable serum levels of mMCP-1 after the oral food challenge (Figure 3E). Both N-OVA- and D-OVA-sensitized groups had the highest serum levels of mMCP-1 after the oral food challenge (compared to the PBS control group; *p* < 0.05). Statistical differences in mMCP-1 serum levels were not found among the food-challenged groups (*p* > 0.05) (Figure 3E). Interestingly, the anaphylactic shock score and mMCP-1 serum levels between the intragastric- and oral food-challenged groups were similar (*p* > 0.05 for both evaluations).

## 4. Discussion

Although the IP route performs better than the intragastric one for inducing consistent and reproducible N-OVA-specific IgE responses [10], the specificity of the antibodies induced could vary depending on the structural state of the antigen administered, as demonstrated for anti-OVA IgG antibodies [7,8]. Triggering allergic reactions to proteins requires the recognition of the same allergenic antigen by two adjacent IgE antibodies, which must be coupled to the FcεRI present in the cell membrane of mast cells. Therefore, IgEs that react with the allergens in the form they are in the food matrix and are recognized by the immune system should be produced by an ideal animal model of food allergy. This concept has relevance because molecular interactions between allergens and other food matrix components occur during food processing and digestion [1,14,15]. Loss of linear and conformational epitopes occurs after digestion, and food processing can lead to the generation of neoepitopes [16]. To be reliable, an animal model of food allergy must trigger allergic reactions readily detected with analytical and non-analytical methods. The present study evaluated the potential to sensitize mice to various structural forms of OVA and to trigger responses after an oral food challenge with a food matrix in the search for an animal model to assess both the allergenic potential of processed food products and the effectiveness of processing techniques to produce hypoallergenic foods.

At least one mouse was sensitized to one of the structural forms of OVA evaluated, triggering IgE responses that recognize both N-OVA and D-OVA. In all groups, the IgE responses to both N-OVA and D-OVA were inconsistent, although more robust against N-OVA. In line with these findings, Bøgh et al. reported that after IP administration, native and denatured cow’s milk allergens (β-lactoglobulin and α-lactalbumin) triggered different sensitizing capacities, with native allergens sensitizing more effectively than denatured ones [17]. These results, and the ones reported in the present study, suggest that mice can be IP sensitized to different structural forms of food allergens, and this fact should be considered in the search for an animal model to evaluate the allergenic potential of processed foods. Furthermore, it should be highlighted that undetectable IgE levels using ELISA do not mean the absence of sensitization, as in our experience, serum levels of biological markers of an allergic immune response can become evident after an intragastric challenge [10]. In the present study, we used Al(OH)_3_ (Imject Alum) for OVA-sensitization, a systemic adjuvant that enhances the production of both IgG and IgE antibodies [17,18], which can compete for the same OVA epitopes [19]. In fact, a high ratio of IgG/IgE in serum after IP sensitizations [20,21] as well as the convenience of depleting IgG to accurately assess IgE in serum [11] were documented. Protocols were recently published for this purpose [21]. Thus, the lack of IgE reactivity evaluated using ELISA can be explained by IgG epitope masking, as stated by others [11,22]. Certainly, our findings cannot be totally extrapolated to humans since the underlying mechanisms of sensitization and elicitation in humans and mice are not completely understood [23]. However, individuals with very low allergen-specific IgE levels can trigger IgE-mediated allergic manifestations [24,25]. These individuals have been categorized as IgE responders with high specific activity [26]. Therefore, it has been suggested that the specific activity of IgE be assessed before we rule out a case of allergy rather than relying solely on the serum levels of IgE [26,27].

In that context, the ability of OVA-sensitized mice to trigger an allergic reaction was evaluated. As proof of the specific activity of the anti-OVA IgE, serum levels of mMCP-1 and signs of an allergic response were evaluated after the mice underwent intragastric challenges with a N-OVA solution. Despite the null or very low IgE reactivity to N-OVA, anaphylactic shock signs and mMCP-1 serum levels were evident in all the OVA-sensitized and intragastrically challenged groups. mMCP-1 is a biological marker of an allergic response in murine food allergy models as it is strongly associated with mast cell degranulation [5,28]. Therefore, the increase in mMCP-1 serum levels after the intragastric challenge with N-OVA shows both that the sensitization protocol can trigger an anti-N-OVA IgE response with biological activity and that these IgEs can recognize OVA epitopes that survive their passage through the gastrointestinal tract.

Protein lability to pepsin digestion is a parameter related to allergenicity [9,29,30]. Resistance to gastric digestion is a characteristic shared by some major allergens [31,32], but an allergic reaction or sensitization is unlikely to occur in the stomach due to its thick mucus layer, low antigen sampling, and lack of absorptive properties [33]. However, digested contents, which carry allergens released during early gastric digestion, come into contact with immune cells in the small intestine and can promote sensitization or allergic reactions [34,35,36]. In this context, despite their lability to gastric digestion, allergenic OVA peptides can survive such digestion [37]. In fact, studies in BALB/c mice sensitized to OVA through different routes (oral, intraperitoneal, and intradermal) showed that T and B cell epitopes can remain immunologically relevant after OVA administration [38]. Further in silico studies showed that linear B cell epitopes (#aa, ^55^KVVRFD^60^; ^277^KIKVYL^282^) and T cell ones (#aa, ^323^ISQAVHAAHAEINEAGR^339^) can be generated after pepsin digestion [39]. These epitopes are contained within allergenic fragments generated after IP sensitization to OVA [37]. In line with the previous findings, in the present study, mice sensitized to OVA peptides obtained after pepsin digestion triggered allergic responses similar to those observed in other OVA-sensitized groups. This suggests that irrespective of the structural state of OVA, IP sensitization induces the production of IgE antibodies that recognize OVA allergenic epitopes that remain after gastrointestinal digestion when OVA is administered orally in an isolated form.

Food processing conditions could promote interactions between OVA and other matrix components, and these interactions can change the allergenic potential of OVA [1,3,15]. Furthermore, gastrointestinal digestion could also change the characteristics of allergens and the way they interact with the immune system, impacting their allergenicity [2]. To address this issue, a sensitive animal model of food allergy is crucial. The model should trigger an allergic response after the consumption of food matrices containing the allergen of interest. This approach could be a key tool to evaluate how processing methods or the food matrix composition can affect the threshold for triggering an allergic response. In this sense, all OVA-sensitized mice underwent a food challenge using a cookie formulated with OVA as a model of a food matrix. Most mice challenged developed mild to severe anaphylactic shock signs after cookie consumption. Excluding the group sensitized with FK-OVA, all OVA-sensitized groups showed significantly higher mMCP-1 serum levels than the control group. Notably, after we had quantified the total cookie consumption, we found that the OVA dose used to trigger the allergic response in the food challenge was between 6.9 and 20 times lower (0.1–0.29 mg of OVA) than the one used for the intragastric challenge (2.0 mg of native OVA). The lower OVA doses required to trigger an allergic response after the food challenge could be a consequence of either the sensitivity of the murine OVA allergy model or potential interactions between the food matrix and OVA allergens that may enhance the allergic response to some degree. For instance, solid foods (like cookies) can protect allergens from the acid environment of the stomach due to their delayed dissolution and subsequent allergen release [40,41]. Additionally, carbohydrate-rich matrices can act as a physical barrier, inhibiting allergen digestion and influencing the production of digested contents with immunologically relevant allergens [42]. Furthermore, the presence of polysaccharides in food matrices can enhance IgE reactivity against OVA [43]. In a baked glutinous food matrix, OVA exhibits almost complete resistance after 120 min of pepsin digestion, and after 60 min of duodenal digestion, immunoreactive polypeptides are generated [44]. Despite these findings, the methodology used in the present study does not allow for a thorough explanation of the low OVA doses needed to trigger an allergic response after an oral food challenge but contributes to our understanding in the search for a standardized animal model to evaluate the allergenic potential of food matrices. Future studies employing the proposed egg allergy model to evaluate the influence of food matrices on the allergic response are warranted.

## 5. Conclusions

The present work shows that BALB/c mice can be sensitized IP to OVA in different structural conformations. Although the serum levels of anti-OVA IgE cannot be detected in all cases using ELISA, food challenges are recommended to confirm sensitization and evaluate the allergenic potential of food matrices. Modifying the structural state of OVA to evaluate the allergic response to this protein in baked foods can be unnecessary as the animal model of OVA allergy efficiently triggers an allergic response to N-OVA in a food matrix, and this is independent of the structural state of OVA to carry out IP sensitizations. The murine model of egg allergy proposed in this study holds potential for evaluating the impact of food matrix composition and processing on the threshold of egg-allergic responses.

## Data Availability

All data are available in the manuscript.
